# The importance of selecting the appropriate reference genes for quantitative real time PCR as illustrated using colon cancer cells and tissue

**DOI:** 10.12688/f1000research.7656.2

**Published:** 2016-03-09

**Authors:** Catríona M. Dowling, Dara Walsh, John C. Coffey, Patrick A. Kiely

**Affiliations:** 1 Department of Life Sciences, and Materials and Surface Science Institute, University of Limerick, Limerick, Ireland; 2 Health Research Institute, University of Limerick, Limerick, Ireland; 3 Graduate Entry Medical School, University of Limerick, Limerick, Ireland; 4 4i Centre for Interventions in Infection, Inflammation and Immunity, Graduate Entry Medical School, University of Limerick, Limerick, Ireland

**Keywords:** Quantitative real-time PCR, Normalisation, Reference Genes, NormFinder, Colon Cancer

## Abstract

Quantitative real-time reverse-transcription polymerase chain reaction (RT-qPCR) remains the most sensitive technique for nucleic acid quantification. Its popularity is reflected in the remarkable number of publications reporting RT-qPCR data. Careful normalisation within RT-qPCR studies is imperative to ensure accurate quantification of mRNA levels. This is commonly achieved through the use of reference genes as an internal control to normalise the mRNA levels between different samples. The selection of appropriate reference genes can be a challenge as transcript levels vary with physiology, pathology and development, making the information within the transcriptome flexible and variable. In this study, we examined the variation in expression of a panel of nine candidate reference genes in HCT116 and HT29 2-dimensional and 3-dimensional cultures, as well as in normal and cancerous colon tissue. Using normfinder we identified the top three most stable genes for all conditions. Further to this we compared the change in expression of a selection of PKC coding genes when the data was normalised to one reference gene and three reference genes. Here we demonstrated that there is a variation in the fold changes obtained dependent on the number of reference genes used. As well as this, we highlight important considerations namely; assay efficiency tests, inhibition tests and RNA assessment which should also be implemented into all RT-qPCR studies. All this data combined demonstrates the need for careful experimental design in RT-qPCR studies to help eliminate false interpretation and reporting of results.

## Introduction

Gene expression analysis is a critical and important tool in molecular diagnostics and medicine ^1– 4^. Quantification of RNA transcripts is carried out using one of four common methods; reverse transcription polymerase chain reaction (RT-PCR) ^5^, RNase protection assays ^6^, northern blotting and *in situ* hybridisation ^7^, and less commonly now using cDNA arrays ^8^. At present, the most popular and widely used method for gene expression is fluorescence based quantitative real time PCR (RT-qPCR) ^9^. It is the most sensitive and flexible of the quantitative methods with a capacity to detect and measure minute amounts of nucleic acids ^10, 11^. There are two types of quantitative methods that can be applied within RT-qPCR; absolute quantification and relative quantification. Absolute quantification relates the PCR signal to a standard curve to determine the input copy number of the gene of interest. In contrast, relative quantification evaluates the change in expression of a target gene relative to a reference group, for example an untreated control ^12^.

When employing RT-qPCR to compare mRNA levels between two different test conditions, it is imperative that reference genes are utilised carefully ^9, 10^. Normalisation of the data with these reference genes is essential for correcting results of different amounts of input RNA, uneven loading, reverse-transcription yield, efficiency of amplification and variation within experimental conditions ^9, 13^. The mRNA of reference genes should be stably expressed and their expression should not be affected by experimental condition or by any human disease ^14^. Numerous studies have demonstrated that common reference genes, such as β-Actin and GAPDH, which are largely accepted as being stably expressed within cells, can in fact show large variations in expression ^15– 18^. Despite the awareness that validation of the stability of reference genes is an essential component for accurate RT-qPCR analysis, this consideration is still largely disregarded ^19– 21^.

Further to this, it is reported that over 90% of gene expression analysis published in high impact journals used only one reference gene ^22– 24^. It has since been widely documented that normalisation of data with a single reference gene can lead to inaccurate interruption of results ^10, 25, 26^. Taken together, this highlights the importance of selecting the optimal number and type of reference genes for any RT-qPCR study. Other essential considerations such as; analysis of assay efficiency, testing for inhibition with biological samples and reporting the quality and integrity of input RNA are all highlighted in the ‘MIQE Guidelines: *M*inimum *I*nformation for Publication of *Q*uantitative Real-Time PCR *E*xperiments’ ^10^.

In this study, we sought to highlight the importance of carefully-designed RT-qPCR studies in order to avoid the reporting of inaccurate and misleading information. We test a panel of nine candidate reference genes and report their stability between 2-dimensional and 3-dimensional HCT116 and HT29 colon cancer cell lines, as well as between normal and cancerous tissue from colon cancer patients. We also demonstrate useful tests that should be implemented within RT-qPCR studies to ensure that studies comply with the MIQE guidelines.

## Methods

### Cell culture

HCT116 (ATCC ^®^ CCL-247™) and HT29 (ATCC ^®^ HTB-38™) cell lines were obtained from ATCC. These cell lines were cultured in complete Dulbecco's modified essential medium (DMEM) supplemented with 10% of foetal bovine serum, 1% of penicillin/streptomycin and 1% of L-glutamine. All cells were incubated at 37°C in a humidified 95% air/5% CO _2_ environment. Cellular suspensions were obtained by adding 0.5% trypsin to the cultures and incubating at 37°C at 5% CO _2_.

### 3-dimensional cell cultures

Individual wells of a 6-well plate were coated with Matrigel ^TM^ (BD Biosciences) and placed in an incubator at 37°C for 30 min. Cell lines were trypsinized and counted. 50,000 cells/ml were resuspended in DMEM supplemented with 2% Matrigel ^TM^. Cells were placed in Matrigel ^TM^ coated wells for 30 min at 37°C, after which DMEM supplemented with 2% Matrigel ^TM^ was added to the cultures. Cells were maintained in culture for 6 days in an incubator at 37°C, 5% CO _2_ with fresh medium added every 2 days. On day 6, cultures were harvested using EDTA/PBS and either fixed with paraformaldehyde (PFA) for confocal analysis (Zeiss LSM 710) or used for RNA extraction.

### Clinical samples

Following ethical approval from the University Hospital Limerick’s Ethics Committee (ethical approval number 73/11), tissue samples measuring approximately 0.5cm in diameter were collected from patients undergoing surgery in University Hospital Limerick. Normal tissue from the patients was also collected approximately 10 cm away from the cancer tissue. Specimens were immediately placed in Allprotect tissue reagent (Qiagen) and stored at -80°C.

### RNA extraction and cDNA synthesis

2-dimensional and 3-dimensional cell cultures were trypsinised as described above and frozen tissue was immersed in liquid nitrogen and ground into powder. Lysis buffer was added to the cells and tissue and the samples transferred to tubes using a 21-gauge needle. Total RNA was extracted as per Qiagen RNeasy Mini Kit instructions. RNA was quantified using a Nanodrop Spectrophotometer (Thermo Scientific) and stored at -80 degrees. RNA purity was evaluated by the ratio of absorbance at 260/280 nm and RNA quality was evaluated through visualization of the 28S:18S ribosomal RNA ratio on a 1% non-denaturing agarose gel. Total RNA (1 μg) was synthesised into cDNA using Vilo cDNA synthesis kit (Invitrogen) and stored at -20 degrees.

### Real-time PCR

Real-time PCR was conducted using the ABI 7900 HT instrument (Applied Biosystems) following supplier instructions. Taqman ^®^ Gene Expression Assay Kits (Applied Biosystems) were used to analyse the gene expression of protein kinase c (PKC) coding genes. Data was normalised to either one reference gene or three reference genes (see below).

### Assay efficiency test

The efficiency of each assay was determined by means of a calibration curve with the logarithm of the initial template concentration plotted on the x axis and the Cq plotted on the y axis. The slope of the graph was obtained and the PCR efficiency was calculated using the equation: 10 ^-1/slope^-1.

### Inhibition test

Real-time PCR was conducted on corn DNA using a corn gene assay with a known Cq value of 24–26. Samples of cDNA from 2D and 3D HCT116 and HT29 cultures and from patient tissue was added to the reaction to test for an inhibitory components that may be present in these biological samples.

### Selection of reference genes

All nine reference genes ( Table 1, Table 2) were purchased as pre-designed Taqman ^®^ Gene Expression Assays. The Cq value of each reference gene was determined for all biological samples. Normfinder was used to determine the most stable reference genes between 2D and 3D cell cultures as well as between normal and cancer tissue. Differences in gene expression levels of the PKC coding genes was determined using Pair Wise Fixed Reallocation Randomisation Test ^©^ as per REST ^©^ software. Within the software data was normalised to either the top reference gene or the top three reference genes.

**Table 1.** Description of the nine candidate housekeeper genes used in the study. The accession numbers for each gene are taken from the National Center for Biotechnology Information.

Symbol	Name	Accession Number	Function
**B2M**	Beta 2 Microglobulin	NM_004048	Important cell surface structure
**PMM1**	Phosphomannomutase 1	NM_002676	Synthesis of the GDP-mannose and dolichol-phosphate-mannose
**TBP**	TATA Box Binding Protein	NM_001172085	Transcription factor
**RPLPO**	Large Ribosomal Protein	NM_053275	Ribosomal Protein
**GUSB**	Beta Glucuronidase	NM_000181	Glycoprotein
**PGK1**	Phosphoglycerate Kinase 1	NM_000291	Glycolytic enzyme
**ACTB**	Beta Actin	NM_001101	Cytoskeleton Protein
**PPIA**	Peptidylprolyl Isomerase A (Cyclophilin A)	NM_021130	Catalyses the cis-trans isomerization of proline imidic peptide bonds

**Table 2.** Additional assay information.

Symbol	Assay ID	Exon Boundary	Amplicon size
**B2M**	Hs00187842_m1	1-2	64
**PMM1**	Hs00160195_m1	1-2	111
**TBP**	Hs00427620_m1	2-3	91
**RPLPO**	Hs99999902_m1	3-3	105
**GUSB**	Hs00939627_m1	8-9	96
**PGK1**	Hs00943178_g1	5-6	73
**ACTB**	Hs01060665_g1	2-3	63
**PPIAq**	Hs04194521_s1	5-5	94

## Results

Cq Values for reference genes in HCT116 cell lines The three Cq values for each reference gene is displayed for the 2-dimensional and 3-dimensional HCT116 cell cultures. Click here for additional data file. Copyright: © 2016 Dowling CM et al. 2016 Data associated with the article are available under the terms of the Creative Commons Zero "No rights reserved" data waiver (CC0 1.0 Public domain dedication).

Cq Values for PKC coding genes and reference genes in HCT116 cell lines The three Cq values for each PKC coding gene and the appropriate reference genes is displayed for the 2-dimensional and 3-dimensional HCT116 cell cultures. Click here for additional data file. Copyright: © 2016 Dowling CM et al. 2016 Data associated with the article are available under the terms of the Creative Commons Zero "No rights reserved" data waiver (CC0 1.0 Public domain dedication).

Cq Values for reference genes in HT29 cell lines The three Cq values for each reference gene is displayed for the 2-dimensional and 3-dimensional HT29 cell cultures. Click here for additional data file. Copyright: © 2016 Dowling CM et al. 2016 Data associated with the article are available under the terms of the Creative Commons Zero "No rights reserved" data waiver (CC0 1.0 Public domain dedication).

Cq Values for PKC coding genes and reference genes in HT29 cell lines The three Cq values for each PKC coding gene and the appropriate reference genes is displayed for the 2-dimensional and 3-dimensional HT29 cell cultures. Click here for additional data file. Copyright: © 2016 Dowling CM et al. 2016 Data associated with the article are available under the terms of the Creative Commons Zero "No rights reserved" data waiver (CC0 1.0 Public domain dedication).

Cq Values for reference genes in normal and colon cancer tissue The three Cq values for each reference gene is displayed for the normal and colon cancer tissue. Click here for additional data file. Copyright: © 2016 Dowling CM et al. 2016 Data associated with the article are available under the terms of the Creative Commons Zero "No rights reserved" data waiver (CC0 1.0 Public domain dedication).

Cq Values for PKC coding genes and reference genes in normal and colon cancer tissue The three Cq values for each PKC coding gene and the appropriate reference genes is displayed for the normal and colon cancer tissue. Click here for additional data file. Copyright: © 2016 Dowling CM et al. 2016 Data associated with the article are available under the terms of the Creative Commons Zero "No rights reserved" data waiver (CC0 1.0 Public domain dedication).

Cq values for sample assay efficiency test The Cq values obtained when HT29 cDNA was serial diluted and the gene PRKCA was amplified. Click here for additional data file. Copyright: © 2016 Dowling CM et al. 2016 Data associated with the article are available under the terms of the Creative Commons Zero "No rights reserved" data waiver (CC0 1.0 Public domain dedication).

Cq values for inhibition assay test The Cq values obtained for the corn assay when each of the sample types indicated are added to the RT-qPCR. Click here for additional data file. Copyright: © 2016 Dowling CM et al. 2016 Data associated with the article are available under the terms of the Creative Commons Zero "No rights reserved" data waiver (CC0 1.0 Public domain dedication).

### Comparison of reference genes in HCT116 2-dimensional and 3-dimensional cultures

**Figure 1.**
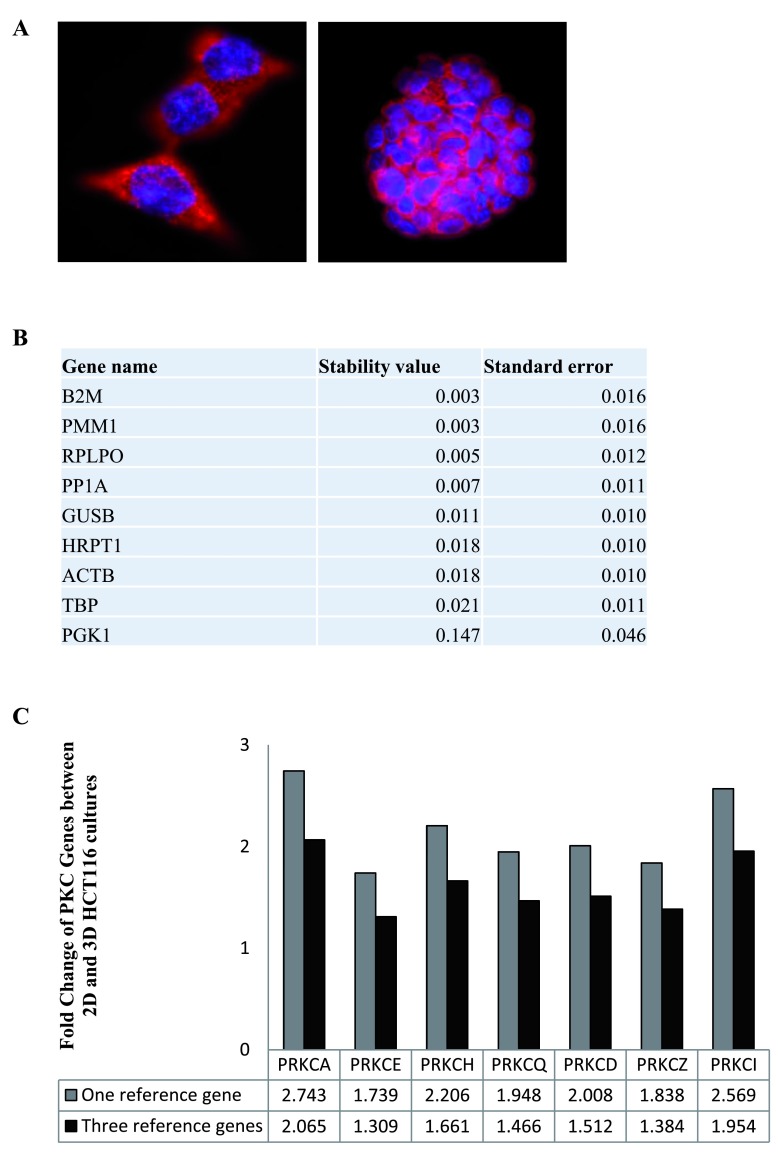
Reference genes in 2-dimensional and 3-dimensional HCT116 cultures. The stability of the nine candidate reference genes between 2D and 3D HCT116 cultures was analysed using NormFinder. ( **A**) Immunofluorescence images of HCT116 cells in 2D (100X) **(left panel)** and 3D **(right panel)** cell cultures (63X). ( **B**) Table displaying the stability levels of the nine candidate reference genes between the 2D and 3D cultures. ( **C**) Graph representing the fold change of PKC coding genes in 3D cultures compared to 2D cultures when using one reference gene (B2M) versus three reference genes (B2M, PMM1 and RPLPO).

In this study, we wanted to compare and validate the stability of reference genes used in quantitative real time PCR (RT-qPCR). To do this, HCT116 cells were grown in 2-dimensional and 3-dimensional cultures ( Figure 1A). Following this, RNA was extracted from the cultures and cDNA was synthesised. Quantitative real time PCR was utilised to measure the variability in RNA transcript levels of 9 reference genes (RG) ( Table 1) in the 2-dimensional and 3-dimensional cultures. The expression levels of the candidate reference genes were determined using the raw Cq values and NormFinder was then utilised to verify the stability of the genes. Normfinder ranks the RGs according to their stability values under the tested conditions. The top three stable genes when comparing 2-Dimensional and 3-dimensional HCT116 cultures were B2M, PMM1 and RPLPO, with B2M and PMM1 showing identical stability levels ( Figure 1B, Dataset 1). Next, we wanted to elucidate the benefit of normalising data to more than one RG. To do this, we compared the expression of seven PKC coding genes in 3-Dimensional HCT116 cultures compared to 2-dimensional HCT116 cultures. The data was normalised to either one RG, B2M, or normalised to three RGs, B2M, PMM1 and RPLPO ( Figure 1C, Dataset 2). Results indicate that using one RG gives fold changes that are greater than the fold changes obtained using three RGs.

### Comparison of reference genes in HT29 2-dimensional and 3-dimensional cultures

**Figure 2.**
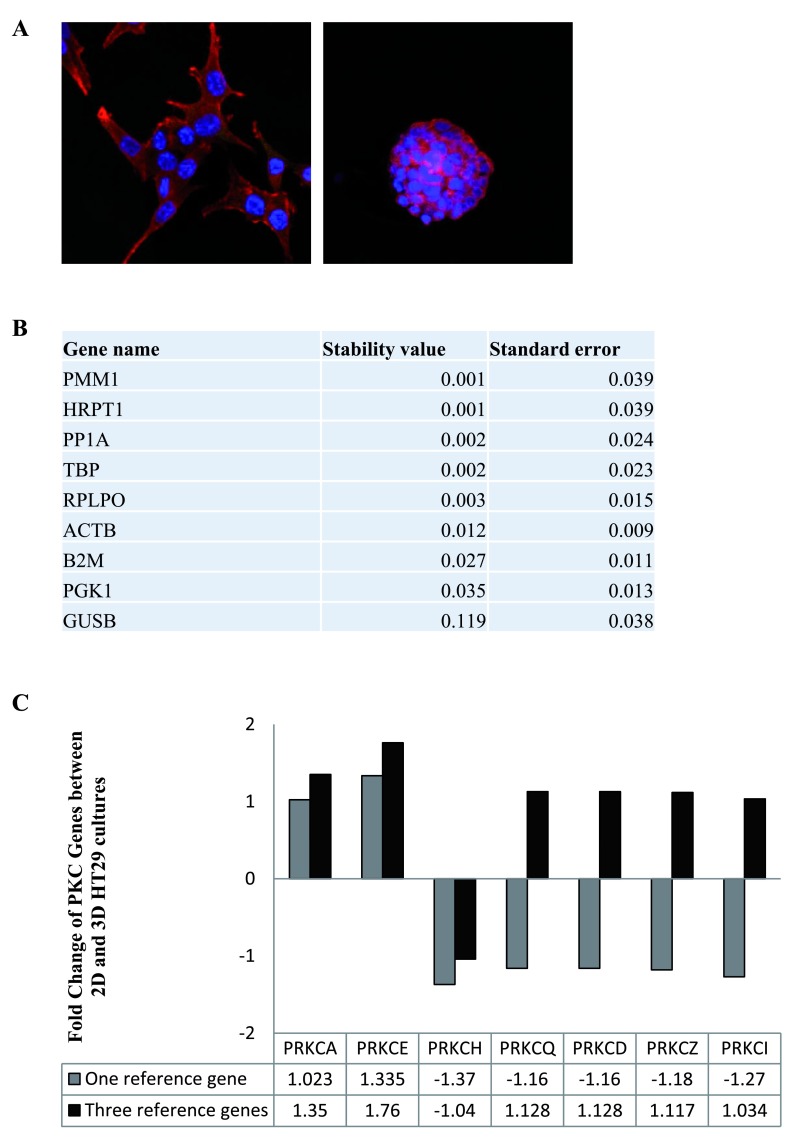
Reference genes in 2-dimensional and 3-dimensional HT29 cultures. The stability of the nine candidate reference genes between 2D and 3D HT29 cultures was analysed using NormFinder. ( **A**) Immunofluorescence images of HT29 cells in 2D (100X) **(left panel)** and 3D **(right panel)** cell cultures (63X). ( **B**) Table displaying the stability levels of the nine candidate reference genes between the 2D and 3D cultures. ( **C**) Graph representing the fold change of PKC coding genes in 3D cultures compared to 2D cultures when using one reference gene (PMM1) versus three reference genes (PMM1, HRPT1 and PP1A).

Next, we compared the stability of the same 9 candidate reference genes in HT29 cultures. The cells were grown in 2-dimensional and 3-dimensional cultures ( Figure 2A) before using RT-qPCR to determine the stability of the RGs between the two conditions. Normfinder revealed the most stable RGs were PMM1, HRPTI, PP1A and TBP ( Figure 2B, Dataset 3) with PMM1 and HRPTI having a value of 0.001 and PP1A and TBP having a value of 0.002. Again, we examined the expression of the PKC coding genes in the cultures and normalised the data to one RG, PMM1, or three RGs, PMM1, HRPTI and PP1A ( Figure 2C, Dataset 4). Our results indicate that there is variation in the fold changes obtained when using one RG versus three RGs. In some instances, genes that are found to be down-regulated when normalising with one RG are in fact up-regulated when normalising with three RGs.

### Comparison of reference genes in normal colon tissue versus colon cancer tissue

**Figure 3.**
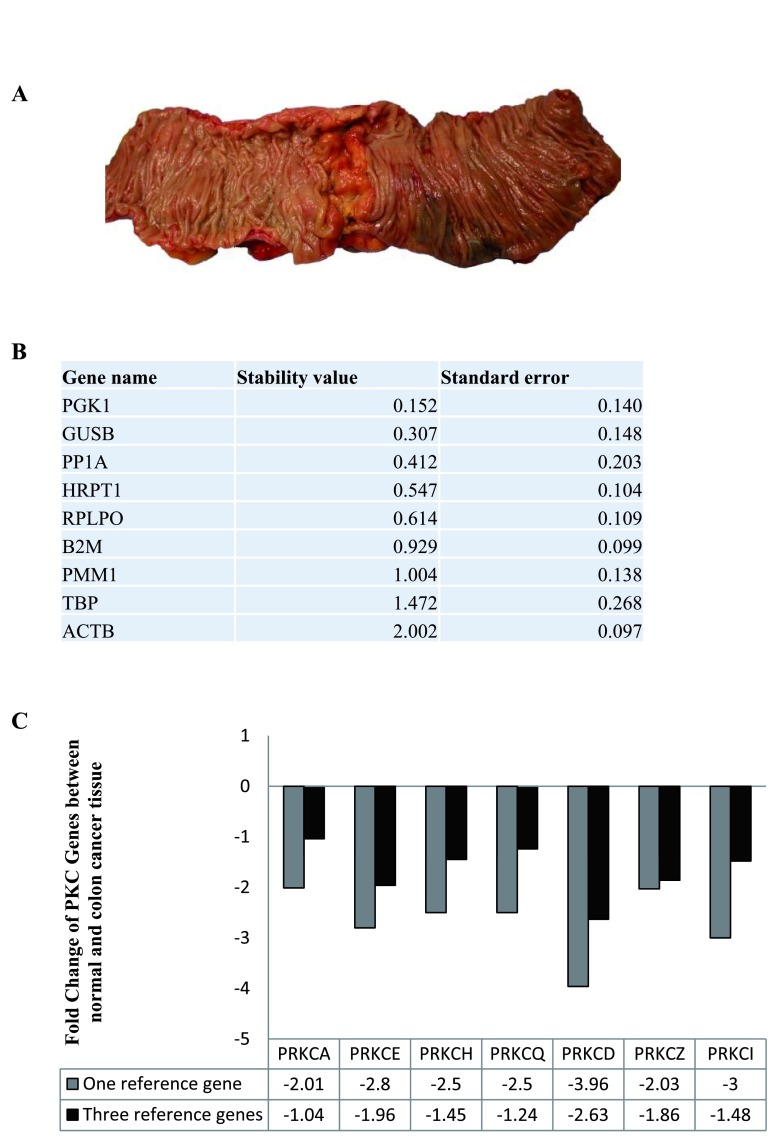
Reference genes in normal and colon cancer tissue. The stability of the nine candidate reference genes between normal and cancer tissue was analysed using NormFinder. ( **A**) Surgical image of specimen resected from a colon cancer patient. ( **B**) Table displaying the stability levels of the nine candidate reference genes between the normal and cancer tissue. ( **C**) Graph representing the fold change of PKC coding genes in cancer tissue compared to normal tissue (n=21) when using one reference gene (PGK1) versus three reference genes (PGK1, GUSB and PP1A).

Following this, we wanted to examine the stability of the nine candidate RGs in normal and colon cancer tissue. We used fresh tissue samples that were excised from both the cancer tissue and normal distant tissue of individual patients ( Figure 3A). As above, the expression levels of the nine candidate RGs were determined and Normfinder was used to establish the stability of the genes. PGK1, GUSB and PP1A were ranked as the most stable genes between normal and cancerous tissue ( Figure 3B, Dataset 5). Next, we examined the change in PKC coding genes in colon cancer tissue when the data was normalised to one RG, PGK1, and normalised to three RGs, PGK1, GUSB and PP1A ( Figure 3C, Dataset 6). The results demonstrate that using one RG can present fold changes that are up to 2-fold greater than when using three RGs.

### Considerations when conducting RT-qPCR

**Figure 4.**
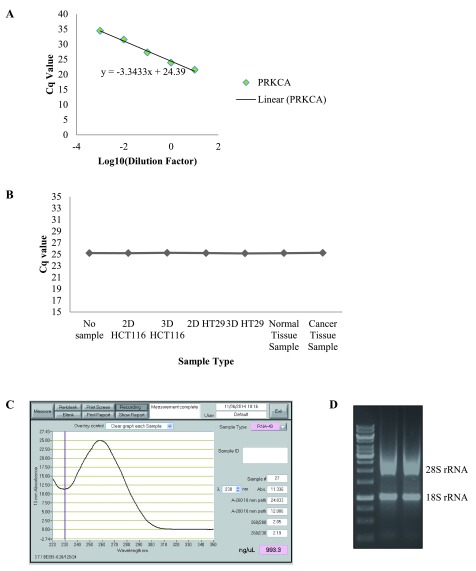
Considerations to comply with during RT-qPCR. ( **A**) Representative graph of assay efficiency check. ( **B**) Graph representing the inhibition test for all biological samples. ( **C**) Representative graph from Nanodrop Spectrophotometer displaying the quantity and purity of the RNA. ( **D**) Representative image of non-denaturing agarose gel displaying the 28S:18S ribosomal RNA ratio for RNA samples.

Taken together, the results indicate that variations in fold changes can occur depending on the RG used to normalise data; making the selection of the correct RGs an imperative part of RT-qPCR studies. Further to this, the testing and reporting of assay efficiency is also essential to prevent the reporting of misinformation. Taking this into consideration, we examined the efficiency of all the RG assays and PKC coding genes assays ( Figure 4A, Dataset 7). This information was inputted into the REST ^©^ software when establishing changes in gene expression between tested conditions. Another important consideration when designing RT-qPCR studies is the testing of your cDNA for any contaminants which could lead to the inhibition of the RT-qPCR reaction. For this reason, we added our samples to a standard RT-qPCR reaction using corn DNA and a gene that is known to have a Cq value of 24–26. If there were contaminants present in our cDNA samples this would inhibit the reaction resulting in a reduction in the Cq values ^10^. However, we found no change in the Cq values for the reactions with the cDNA added, indicating the samples do not have any contaminates that will affect the amplification of our genes ( Figure 4B, Dataset 8). It is also essential to report the quality assessment of the RNA templates, such as the RNA quantity, quality and integrity. We evaluated the RNA purity by the ratio of absorbance at 260/280 nm and RNA quality was assessed through visualization of the 28S:18S ribosomal RNA ratio on a 1% non-denaturing agarose gel ( Figure 4C,D).

## Discussion

The first publications using fluorescence-based quantitative real time PCR (RT-qPCR) ^27– 30^ emerged almost a decade ago and since this time it has become the leading technique for gene expression analysis ^31, 32^. While RT-qPCR remains the most sensitive method for the detection of RNA transcripts ^33^ there are also many challenges associated with the technique ^34, 35^. One of the major difficulties is the selection of appropriate reference genes for the normalisation of data. Hence the purpose of this study was to evaluate the stability in expression of nine candidate reference genes in two colon cancer cell lines as well as in normal and cancerous tissue from colon cancer patients. To help find the most suitable reference genes we selected genes which display a variation of functions within cells ( Table 1).

Firstly, we examined the stability of the nine candidate reference genes between 2-dimensional and 3-dimensional HCT116 and HT29 cultures ( Figure 1A, Figure 2A). The use of 3-dimensional cell cultures as cancer models is becoming increasingly popular ^36– 38^; making the availability of appropriate reference genes important to help reduce the reporting of misinformation. When we examined the variation in expression between 2-dimensional and 3-dimensional HCT116 cells we found B2M, RPLPO and PMM1 to be the most stable genes between these two conditions ( Figure 1B). Many publications have highlighted the problems associated with normalisation of data using only one reference gene ^22, 23^, for this reason we wanted to investigate differences in fold changes associated with normalising data to one reference gene compared to three reference genes. To do this, we investigated the change in expression in a selection of protein kinase c (PKC) coding genes between 2-dimensional and 3-dimensional HCT116 cultures. We examined PKCs as they are a group of proteins that are extensively studied for their role in oncogenic signalling ^39^. Interestingly, when normalising the data to the reference gene B2M alone we found the change in expression of PKC coding genes was greater compared to normalisation with the reference genes, B2M, RPLPO and PMM1 together ( Figure 1C). This finding highlights the need for normalisation with more than one reference gene to help eliminate the misinterpretation of fold changes in target genes.

Next we wanted to establish the stability of these reference genes in 2-dimensional and 3-dimensional HT29 cultures ( Figure 2A). Normfinder ranked PMM1, HRPTI, PP1A and TBP as the most stable genes between these cultures ( Figure 2B). It is important to note that despite the fact the treatments here were the same; there was a difference in the selected reference genes for HCT116 and HT29 cultures. This again emphasises the need to conduct stability tests on a panel of reference genes prior to all RT-qPCR studies to ensure data is normalised correctly. Again, we examined the difference in fold changes of PKC coding genes when normalising with varying numbers of reference genes. Importantly, we found that some target genes showing a down regulation when normalised with PMM1 showed no change when normalised to PMM1, HRPT1 and PP1A ( Figure 2C).

RT-qPCR is the most common method used for the quantification of individual genetic differences in normal versus cancerous tissue ^9, 34^. Recent publications demonstrated that 97% of RT-qPCR studies conducted on colorectal cancer contained information that was unreliable ^21^. Thus, when examining difference in mRNA levels between normal and diseased tissue it is imperative the correct reference genes are used to normalise the data to prevent the presence of misleading information in the literature. Using normal and cancer tissue from CRC patients ( Figure 3A) we examined the stability of the nine candidate reference genes, finding PGK1, GUSB and PP1A to be the most stably expressed ( Figure 3B). As before, we compared the expression of PKC coding genes in normal and cancer tissue with the data normalised to either PGK1 alone or PGK1, GUSB and PP1A together. Strikingly we found that using only one reference gene results in a fold change that is up to 2 fold greater than when using three reference genes. This is a very important observation as it clearly displays that the misuse of reference genes could lead to the incorrect reporting of a dysregulated genes in cancerous tissue.

Although the selection of the correct reference genes is a key challenge when conducting RT-qPCR studies there are other aspects of experimental design that also need to be considered ^10^. In this study, we highlighted appropriate tests to comply with necessary measures for RT-qPCR studies ( Table 3). When utilising relative quantification it is essential that the gene assay of the reference gene and the target gene are amplified with comparable efficiencies ^34^. For this reason, we examined the efficiency of all gene assays using a calibration curve ( Figure 4A) and we used this value when evaluating the fold change between conditions. Another important consideration in experimental design is establishing the presence or absence of biological contaminants in samples which may inhibit the RT-qPCR reaction ^40^. We designed an inhibition assay test and displayed that there was no inhibitors present in any of the samples ( Figure 4B). Finally, the documenting of the quality assessment of RNA templates is critical within RT-qPCR studies as it has been observed that there is a difference in gene expression stability between intact and degraded RNA samples from the same tissue and higher gene-specific variation in degraded samples ^34, 41^. In this study, we documented the RNA purity by the ratio of absorbance at 260/280 nm and RNA quality through visualization of the 28S:18S ribosomal RNA ratio on a 1% non-denaturing agarose gel ( Figure 4C,D).

**Table 3.** Checklist of tests to conduct when designing RT-qPCR studies.

Checklist	Suggested Test
**Correct reference genes**	Test a panel of candidate reference genes using Normfinder
**Efficiency of primer** **assays**	Conduct a calibration curve and use the slope of the graph to calculate PCR efficiency with the following equation: 10 ^-1/slope^-1
**Inhibition within samples**	Add samples to a standard RT-qPCR reaction and look for changes in the Cq values
**RNA purity**	Measure the ratio of absorbance at 260/280 nm
**RNA integrity**	Visualization of the 28S:18S ribosomal RNA ratio on a 1% non-denaturing agarose gel

Our data clearly demonstrates that the variability in the expression of reference genes can lead to false interpretation of results; making the selection of the correct genes essential when normalizing RNA concentrations in RT-qPCR analyses. Further to this we have demonstrated appropriate tests to create studies which comply with the MIQE guidelines. The implementation of these guidelines ^10, 42^ should be employed by all reviewers when accepting gene expression studies for publication as it will help eliminate the reporting of inaccurate and misleading information.

## Data availability

The data referenced by this article are under copyright with the following copyright statement: Copyright: © 2016 Dowling CM et al.

Data associated with the article are available under the terms of the Creative Commons Zero "No rights reserved" data waiver (CC0 1.0 Public domain dedication). 

*F1000Research*: Dataset 1. Cq Values for reference genes in HCT116 cell lines, 10.5256/f1000research.7656.d115637 ^43^

*F1000Research*: Dataset 2. Cq Values for PKC coding genes and reference genes in HCT116 cell lines, 10.5256/f1000research.7656.d115638 ^44^

*F1000Research*: Dataset 3. Cq Values for reference genes in HT29 cell lines, 10.5256/f1000research.7656.d115639 ^45^

*F1000Research*: Dataset 4. Cq Values for PKC coding genes and reference genes in HT29 cell lines, 10.5256/f1000research.7656.d115640 ^46^

*F1000Research*: Dataset 5. Cq Values for reference genes in normal and colon cancer tissue, 10.5256/f1000research.7656.d115643 ^47^

*F1000Research*: Dataset 6. Cq Values for PKC coding genes and reference genes in normal and colon cancer tissue, 10.5256/f1000research.7656.d115641 ^48^

*F1000Research*: Dataset 7. Cq values for sample assay efficiency test, 10.5256/f1000research.7656.d115642 ^49^

*F1000Research*: Dataset 8. Cq values for inhibition assay test, 10.5256/f1000research.7656.d111811 ^50^

## Consent

Written informed consent for publication of their clinical details and clinical images was obtained from the patients.

(Ethical approval number 73/11, University Hospital Limerick, Limerick, Ireland).
