# Supplementary material for: The importance of selecting the appropriate reference genes for quantitative real time PCR as illustrated using colon cancer cells and tissue
Source: F1000Res. 2016 Mar 9;5:99. Originally published 2016 Jan 22. [Version 2] doi: 10.12688/f1000research.7656.2 (PMC4768652; doi:10.12688/f1000research.7656.2)
Supplement: Peer review history

# Peer review: Referee response for version 1

**DOI:** 10.5256/f1000research.8245.r12476

**Published:** 2016-02-24

**Authors:** Verónica Ayllón Cases

**Affiliations:** 1 Gene Regulation, Stem Cells and Development Group, Department of Genomic Oncology, Centre for Genomics and Oncological Research (Genyo), Pfizer-University of Granada-Regional Government of Andalusia, Granada, Spain

This article by Dowling *et al*. demonstrates the importance of choosing the right reference genes (RG) when performing RT-qPCR experiments. They have compared the effect of using a single RG versus three RGs on the gene expression values of the interrogated genes on a given experiment. They present data that confirms that using a single RG usually gives greater changes in gene expression than when using a panel of three RGs. As a consequence, many published studies that present RT-qPCR results based on a single RG may have over-estimated gene expression changes and generated misleading results.

As a conclusion of their work, they present a very useful checklist for any researchers that want to perform gene expression analysis using RT-qPCR, which includes all the steps to follow when designing RT-qPCR experiments.

When reviewing this work, there are several minor points that have raised my concern, although they don’t affect the main conclusions of the work. These minor points are:

1. It is not clear to me whether the three Cq values given on the data sets correspond to three independent experiments (biological replicates) or they are three values obtained from the same sample (experimental replicates).
2. In Figure 2C the authors have presented their data in a way that I consider it magnifies their results and it can be slightly misleading. The authors have plotted the fold changes in PKC genes using what is known as “Fold Regulation”, in which the values of Fold Change below 1 are plotted as negative values. When the data is plotted in this way, the area of the graph between +1 and -1 it simply doesn’t exist; the values will always “jump” from >+1 to <-1.
  If the data presented in Figure 2C was plotted without making this conversion to Fold Regulation we will be able to appreciate more clearly that the expression of several PKC genes does not change much – the values will probably oscillate between 1.2 and 0.8.
  My recommendation for the authors is to change the way the present their data in this case where the Fold Regulation data oscillates between positive and negatives values, but they are all very close to 1 (that is, there is only a limited variation in expression relative to the control sample of 2D cultures). I suggest two alternatives:
  - Remove the gap between +1 and -1 in your Y axis.
  - Present your data as Fold Change, without converting it to Fold Regulation.
  As a final comment, also please put your gene names in a way that they don’t overlap with the bars, as it is very difficult to read them. This also applies to Figure 3C.
3. Regarding the assessment of RNA purity and integrity, the authors have used spectrophotometry and running an agarose gel, respectively. This is correct, but if we want to compare RNA integrity across samples it would be better to perform this type of analysis using a Bioanalyzer (Agilent Technologies). With this assay we will be able to obtain a more quantitative measurement of RNA integrity in the form of the RIN value. My suggestion to the authors is, if possible, to complement the data they already have with a Bioanalyzer analysis and the corresponding RIN data. In this way they will confirm that the simpler strategy that they propose is a valid one.

I have read this submission. I believe that I have an appropriate level of expertise to confirm that it is of an acceptable scientific standard.

# Response

**Published:** 2016-03-01

**Authors:** Catríona Dowling

**Affiliations:** University of Limerick, Ireland

We thank the reviewer for the suggestions. We have adjusted the manuscript accordingly and we agree that it is better presented now.

The values given on the datasets corresponds to three independent experiments i.e three biological replicates. We have now written a note within each dataset to clear any ambiguity.

We are grateful to the reviewer for making the observation on Figure 2C. We have now updated the figure and removed the area between 1 and -1. We have also changed the layout in our graphs to ensure the gene names don’t overlap with the bars.

We agree with the reviewer, using a Bioanalyzer is the preferable method for looking at RNA integrity and purity. Unfortunately, it is not possible for us to complement our data with a Bioanalyzer analysis as we don’t have access to this piece of equipment. We used non-denaturing agarose gels and a nanospectrometer for our work.  There are many Universities that do not have access to a Bioanalyzer and for this reason we wanted to offer this alternative approach to encourage all researchers to carry out integrity and purity tests in the absence of such equipment.

# Response

**Published:** 2016-03-05

**Authors:** Catríona Dowling

**Affiliations:** University of Limerick, Ireland

We thank the reviewer for the suggestions. We have adjusted the manuscript accordingly and we agree that it is better presented now.

The values given on the datasets corresponds to three independent experiments i.e three biological replicates. We have now written a note within each dataset to clear any ambiguity.

We are grateful to the reviewer for making the observation on Figure 2C. We have now updated the figure and removed the area between 1 and -1. We have also changed the layout in our graphs to ensure the gene names don’t overlap with the bars.

We agree with the reviewer, using a Bioanalyzer is the preferable method for looking at RNA integrity and purity. Unfortunately, it is not possible for us to complement our data with a Bioanalyzer analysis as we don’t have access to this piece of equipment.

We used non-denaturing agarose gels and a nanospectrometer for our work. There are many Universities that do not have access to a Bioanalyzer and for this reason we wanted to offer this alternative approach to encourage all researchers to carry out integrity and purity tests in the absence of such equipment.

# Peer review: Referee response for version 1

**DOI:** 10.5256/f1000research.8245.r12053

**Published:** 2016-02-02

**Authors:** Gary Loughran

**Affiliations:** 1 School of Biochemistry and Cell Biology, University College Cork, Cork, Ireland

Dowling *et al.* reinforce the necessity of using more than one reference gene (RG) for qRT-PCR. They show that not only should more than one RG be used for normalisation but that a panel of RGs should be tested at an early stage to identify the most stable group. They demonstrate clearly how perilous choosing a single inappropriate RG can produce anomalous data.

While this study was well designed and well performed there are some omissions that would enhance the report by facilitating repetition by others. It would be nice to see a table listing the primer sequences used, expected amplicon size and whether any particular primer pairs are intron spanning. This would be especially useful for the RGs.

One other minor point. Presumably testing the integrity of RNA on a 1% agarose gel was under denaturing conditions (e.g. formaldehyde)?

I have read this submission. I believe that I have an appropriate level of expertise to confirm that it is of an acceptable scientific standard.

# Response

**Published:** 2016-02-05

**Authors:** Catríona Dowling

**Affiliations:** University of Limerick, Ireland

We thank the reviewer for the suggestion.

The sequences of the primers and probes we used in our assays are pre-designed and are the proprietary of Life Technologies who are unable to release this information to us. However, as stated within the MIQE guidelines, it is acceptable to use the unique assay ID for each TaqMan assay in place of the primer and probe sequences. We are grateful to the reviewer for the suggestion to include such information and we will include a table which displays assay ID, exon boundary and amplicon size for all reference genes.

The agarose gels that we ran were non-denaturing gels. We will add this in to the text and to avoid any confusion.
